# The efficacy and prognosis analysis of short-term spinal cord stimulation in the treatment of zoster-associated pain: a retrospective study

**DOI:** 10.3389/fneur.2025.1611447

**Published:** 2025-07-11

**Authors:** Xiang Wang, Yimin Gao, Jing Yu, Wenqu Yang, Wenjing Zhang, Jianzhong Huo

**Affiliations:** ^1^Department of Pain Medicine, Third Hospital of Shanxi Medical University, Shanxi Bethune Hospital, Taiyuan, China; ^2^Department of Gynaecology and Obstetrics, Third Hospital of Shanxi Medical University, Shanxi Bethune Hospital, Taiyuan, China; ^3^Department of Anesthesiology Medicine, Third Hospital of Shanxi Medical University, Shanxi Bethune Hospital, Taiyuan, China; ^4^Orthopedics Department of Shanxi Medical University Second Hospital, Taiyuan, China

**Keywords:** herpes zoster, zoster-associated pain, postherpetic neuralgia, short-term spinal cord stimulation, clinical efficacy, risk factors

## Abstract

**Background:**

Zoster-associated pain (ZAP) is the most common and intractable complication in the clinical setting when patients with herpes zoster (HZ) seek medical treatment. Short-term spinal cord stimulation (stSCS) has been validated as an effective means to relieve ZAP. However, in the existing literature, there is a paucity of comprehensive reports elaborating on the risk factors that impact its treatment efficacy.

**Objective:**

This study aimed to evaluate the clinical efficacy of stSCS in the treatment of ZAP, and to analyze the risk factors that influence the treatment efficacy.

**Methods:**

Clinical data of patients diagnosed with ZAP and who underwent stSCS surgery in the Pain Department of Shanxi Bethune Hospital from January 2020 to December 2023 were collected. A retrospective analysis was performed to evaluate their clinical efficacy. Principal component analysis (PCA) was utilized to screen potential factors influencing the efficacy, and Logistic regression was employed to establish a predictive model. The accuracy of the model was assessed through the Receiver Operating Characteristic (ROC) curve and the C-index.

**Results:**

A total of 98 patients were enrolled in this study. After stSCS treatment, the visual analogue scale (VAS) scores of pain were significantly reduced, the sleep quality of patients was improved, and the dosage of analgesic drugs was markedly decreased compared with that before treatment. The results of multivariate Logistic regression analysis indicated that age [odds ratio (OR): 1.175; 95% confidence interval (CI): 1.864–2.584; *p* = 0.021], disease course (OR:1.894; 95% CI: 1.563–2.365; *p* = 0.003), diabetes mellitus (OR: 2.805; 95% CI: 2.425–3.539; *p* = 0.025), and the pain area size (OR: 3.208; 95% CI: 1.705–2.213; *p* = 0.001) were independent risk factors affecting the efficacy of stSCS in the treatment of ZAP.

**Conclusion:**

Our findings suggest that, stSCS effectively relieves ZAP patients’ pain, reduces analgesics consumption, improves sleep quality, with low complication rate and high safety. Notably, age, disease course, diabetes mellitus, and pain area size are independent risk factors for its efficacy. The C-index and ROC area, both 0.824, show the prediction model has good accuracy and discriminative power.

## 1 Introduction

Zoster-associated pain (ZAP) refers to the neuralgia that occurs during the herpes eruption period and after the herpes healing in patients with herpes zoster (HZ). It is the primary reason for patients to seek medical treatment and one of the most common and intractable complications (1). The exact mechanism of ZAP remains incompletely understood. However, it may be related to the reactivation of the dormant varicella-zoster virus (VZV) lurking in the dorsal root ganglia or cranial ganglia, which causes nerve damage and subsequently sensitizes the peripheral and central nerves, leading to severe pain in the distribution area of the affected nerve (2). Generally, ZAP is classified into three stages according to the disease course: acute herpetic neuralgia (AHN) with a disease course of ≤ 1 month, subacute herpetic neuralgia (SHN) with a disease course of 1–3 months, and postherpetic neuralgia (PHN) with a disease course of > 3 months (3). In Europe, North America, and the Asia-Pacific region, the incidence rate of PHN in HZ patients ranges from 5 to 30% (4). In China, the prevalence rates of HZ and PHN are 7.7 and 2.3% respectively, and the prevalence rate of PHN in HZ patients is 29.8% (5). In some patients, the pain may persist for months or even years after the healing of herpes zoster skin lesions. This not only severely affects patients’ quality of life, sleep, and psychological state, but also may trigger mental disorders such as anxiety and depression, bringing heavy psychological and economic burdens to patients and their families.

At present, the therapeutic modalities for ZAP encompass pharmacological treatment, nerve block, minimally invasive intervention, traditional Chinese medicine and herbal remedies, physical rehabilitation, and other approaches (6). Pharmacological treatment serves as the foundational and predominant means in ZAP management. However, in a significant number of elderly patients, the effectiveness of drug therapy leaves much to be desired. Owing to intolerable adverse reactions, they are compelled to discontinue the medications, consequently leading to persistent and unmitigated pain in clinical practice (7). Research findings have demonstrated that early implementation of minimally invasive interventional surgery can promptly alleviate ZAP and concurrently curtail the incidence of PHN (8). Pulsed radiofrequency (PRF) is recognized as a safe and efficacious treatment option for ZAP. Notwithstanding, for patients with a protracted disease course and severe pain, the durability of its therapeutic effect is rather brief, accompanied by symptom recurrence and suboptimal long-term outcomes (9). Paravertebral nerve block and sympathetic nerve block can assuage the pain manifestations of ZAP patients. Nevertheless, the majority of elderly ZAP patients typically present with comorbidities such as cardiovascular and cerebrovascular disorders and diabetes mellitus, which augment the surgical risks associated with epidural block or sympathetic nerve block and may potentially precipitate postoperative complications like urinary retention, hypotension, or constipation (10). Although a multiplicity of treatment options exists for ZAP, no solitary approach can entirely eradicate the pain experienced by all patients. Even when comprehensive treatment regimens and multimodal combinations are employed, the pain relief achieved in certain patients remains marginal, and they may still endure moderate to severe pain.

Short-term spinal cord stimulation (stSCS), serving as an efficacious neuromodulation modality, entails the implantation of electrodes into the epidural space of the spinal cord. By discharging electrical pulse stimulation toward the dorsal column of the spinal cord, it modulates nerve signal transduction, thus fulfilling the objective of pain alleviation (11). In contrast to the conventional long-term implantable spinal cord stimulation systems, stSCS exhibits several advantages, namely relatively reduced invasiveness, a more abbreviated treatment cycle, and comparatively lower expenditure (12). Over recent years, it has attracted escalating attention and witnessed extensive application in the clinical treatment of ZAP. Prior investigations have demonstrated that stSCS is capable of relieving ZAP and diminishing the incidence of PHN (13, 14). Nevertheless, a certain segment of patients still fails to experience pain relief subsequent to stSCS treatment, particularly those non-acute patients with a protracted disease course. Presently, there is a paucity of research focused on analyzing the prognostic factors associated with stSCS in the treatment of ZAP. This study is designed to probe into the clinical efficacy of stSCS in the treatment of ZAP and dissect the risk factors that impact its efficacy. The overarching goal is to more effectively screen patients who might be responsive to stSCS treatment during the clinical management of ZAP patients, curtail the wastage of medical resources, and furnish scientific underpinnings and practical directives for the more judicious application of stSCS technology in the treatment of ZAP.

## 2 Patients and methods

### 2.1 Patients general information

**Figure 1:**
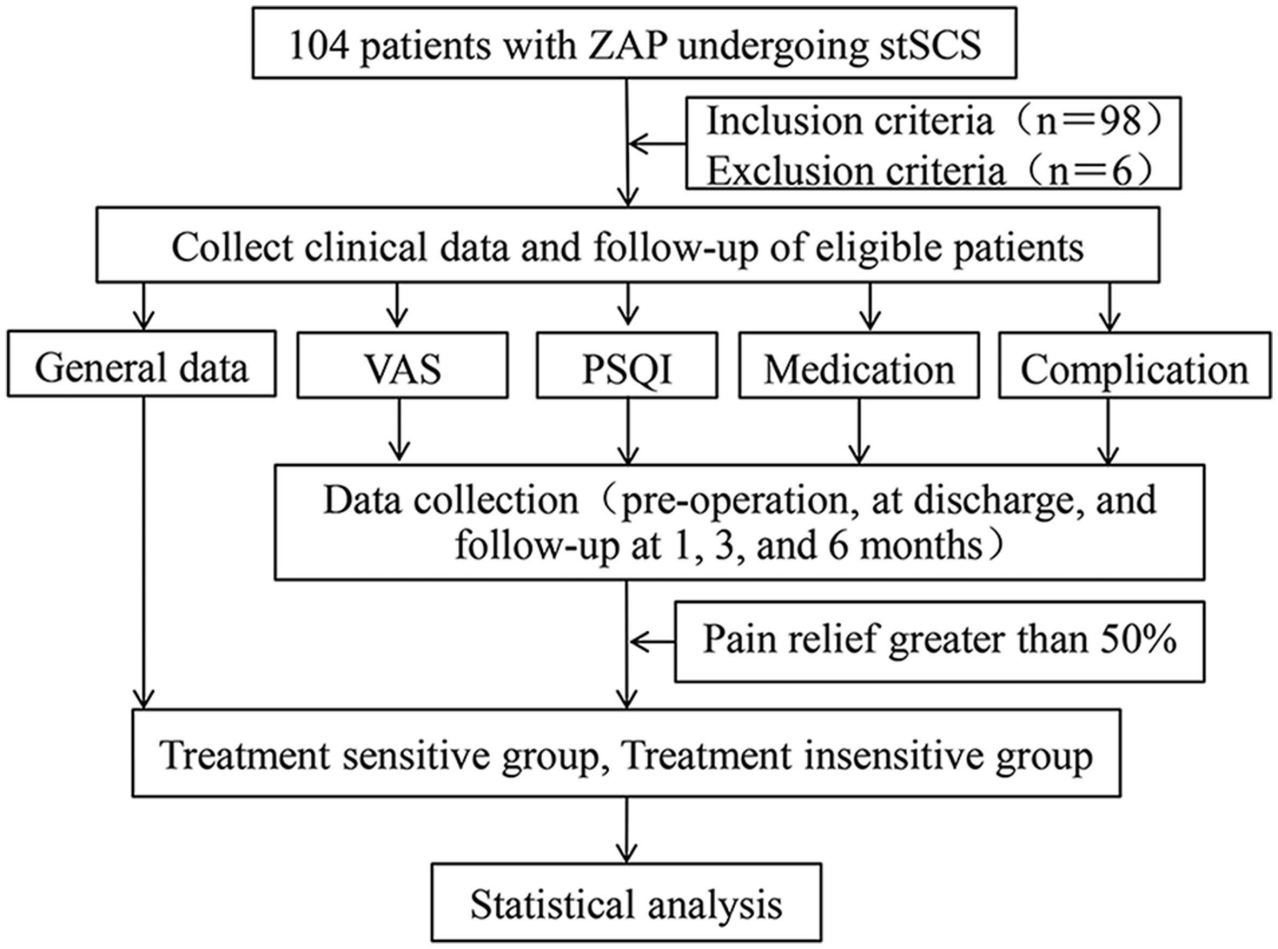
Experimental design and methodology flowchart. ZAP, zoster-associated pain; stSCS, short-term spinal cord stimulation; VAS, visual analogue scale; PSQI, Pittsburgh sleep quality index.

This study retrospectively gathered the clinical data of patients who had been diagnosed with ZAP and had received stSCS surgery in the Pain Department of Shanxi Bethune Hospital spanning from January, 2020, to December, 2023. The general particulars of those patients conforming to the inclusion and exclusion criteria were meticulously organized, and their overall conditions, including blood routine examination results, visual analogue scale (VAS) scores for pain assessment, sleep quality metrics, analgesic drug utilization, and the presence of comorbidities, were thoroughly analyzed. Initially, a total of 104 ZAP patients who had undergone stSCS treatment were sifted through, and ultimately, 98 patients were found to fulfill the inclusion and exclusion benchmarks set by this study. The experimental design flowchart is presented in Figure 1. This study adheres to the ethical precepts stipulated in the Declaration of Helsinki and has obtained the official review and endorsement from the Ethics Committee of Shanxi Bethune Hospital (Ethical Approval Number: YXLL-2025-007).

**Figure 2:**
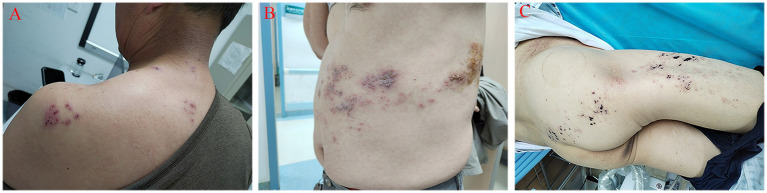
Herpes zoster in different distribution areas. **(A)** herpes zoster in the distribution area of cervical nerves; **(B)** herpes zoster in the distribution area of thoracic nerves; **(C)** herpes zoster in the lumbosacral nerve distribution area.

The cases enrolled in this study comprised patients at the acute, subacute, and chronic stages. The participants incorporated into the research were individuals experiencing pain in the regions innervated by the cervical, thoracic, and lumbar nerves (Figure 2). Following hospitalization, analgesic medications, principally gabapentin and pregabalin, were administered to the patients. Concurrently, a series of comprehensive and pertinent examinations were carried out to eliminate any surgical contraindications. One day before the scheduled surgery, a reassessment of the patients’ pain intensity was conducted. Subsequently, a detailed elucidation of the surgical protocol, potential treatment risks, and conceivable complications was provided to both the patients and their families. Once the informed consent had been obtained from them and the relevant consent forms had been duly signed, the patients were then subjected to the stSCS surgical treatment.

### 2.2 Inclusion and exclusion

The inclusion criteria were as follows: (1) the patients had complete and comprehensive clinical data records; (2) patients suffering from moderate to severe pain, for whom conservative treatment modalities had failed to yield effective results or who were unable to tolerate the adverse drug reactions, specifically, these patients exhibited a VAS score for pain that was equal to or greater than 4 points; (3) those who had been accurately diagnosed with ZAP and subsequently underwent stSCS treatment; (4) the patients who received stSCS treatment, with the stimulation duration extending beyond 2 weeks, enabling them to objectively assess and report their pain experience and successfully complete the follow-up procedures; (5) the herpes lesions were restricted to a single side of the body.

The exclusion criteria were as follows: (1) neuropathic pain caused by other diseases that had the same or overlapping distribution range as ZAP; (2) the duration of electrical stimulation was less than 7 days; (3) the herpes lesions involved the trigeminal nerve region; (4) patients with severe dysfunction of the cardiovascular and cerebrovascular, endocrine, and hematological systems or other severe systemic diseases.

### 2.3 Operation method

Preoperatively, comprehensive and in-depth communication concerning the patient’s medical condition and essential surgical precautions was meticulously carried out with both the patients and their families. This was done to ensure seamless cooperation during the electrode implantation procedure and the subsequent postoperative electrode calibration. The surgical procedures were performed by the same pain specialist. The positioning of the stSCS was contingent upon the specific nerve segments implicated by the herpes infection. Typically, the lower 3 ~ 4 spinal segments relative to the affected nerve segment were chosen as the puncture sites. The entirety of the stSCS operation was executed under the real-time guidance and precise positioning afforded by digital subtraction angiography (DSA).

Upon entering the operating room, the patient was positioned prone on the operating table, with a slender cushion placed beneath the abdomen to optimize comfort and access. Simultaneously, continuous electrocardiogram monitoring and low-flow oxygen supplementation were instituted. By carefully assessing the patient’s pain locus, the corresponding affected nerve segment was accurately identified, and the appropriate interspinous space was selected as the target. The puncture point was precisely designated 0.5 cm lateral to the midline of the spinous process. Following this, routine aseptic disinfection and sterile draping were performed in strict accordance with surgical protocols. Subsequently, local anesthesia was administered using 2 mL of 1% lidocaine. The epidural puncture needle, with its bevel oriented upward, was then carefully inserted along the predetermined puncture point on the affected side. As the needle was advanced through the interlaminar space and penetrated the ligamentum flavum, a distinct loss of resistance was perceptibly felt, and aspiration verified the absence of blood or cerebrospinal fluid. X-ray imaging was promptly employed to confirm the correct placement of the needle tip within the posterior epidural space.

**Figure 3:**
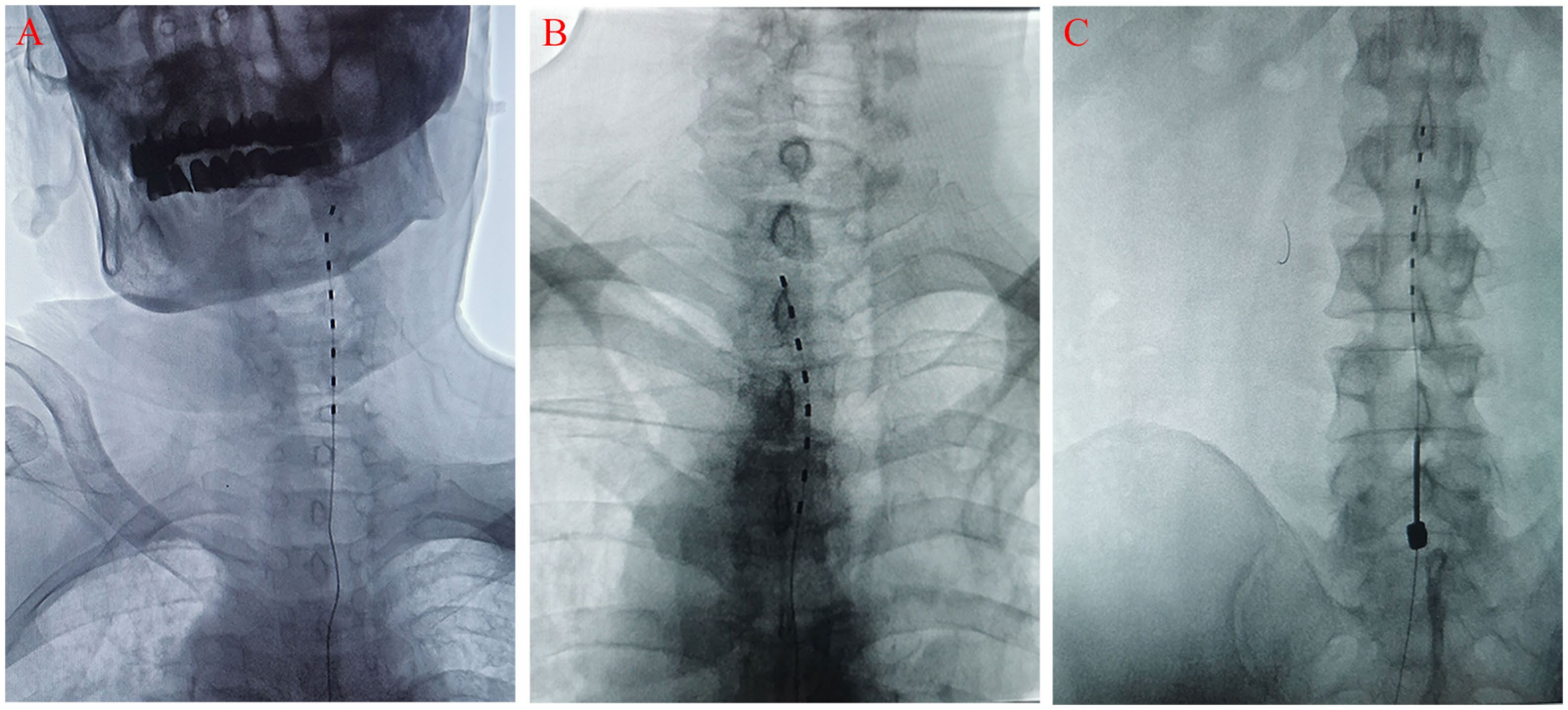
The anteroposterior X-ray film shows that the electrode is located in the target area outside the spinous process and inside the pedicle of vertebral arch. **(A)** neck; **(B)** chest; **(C)** lumbar.

**Figure 4:**
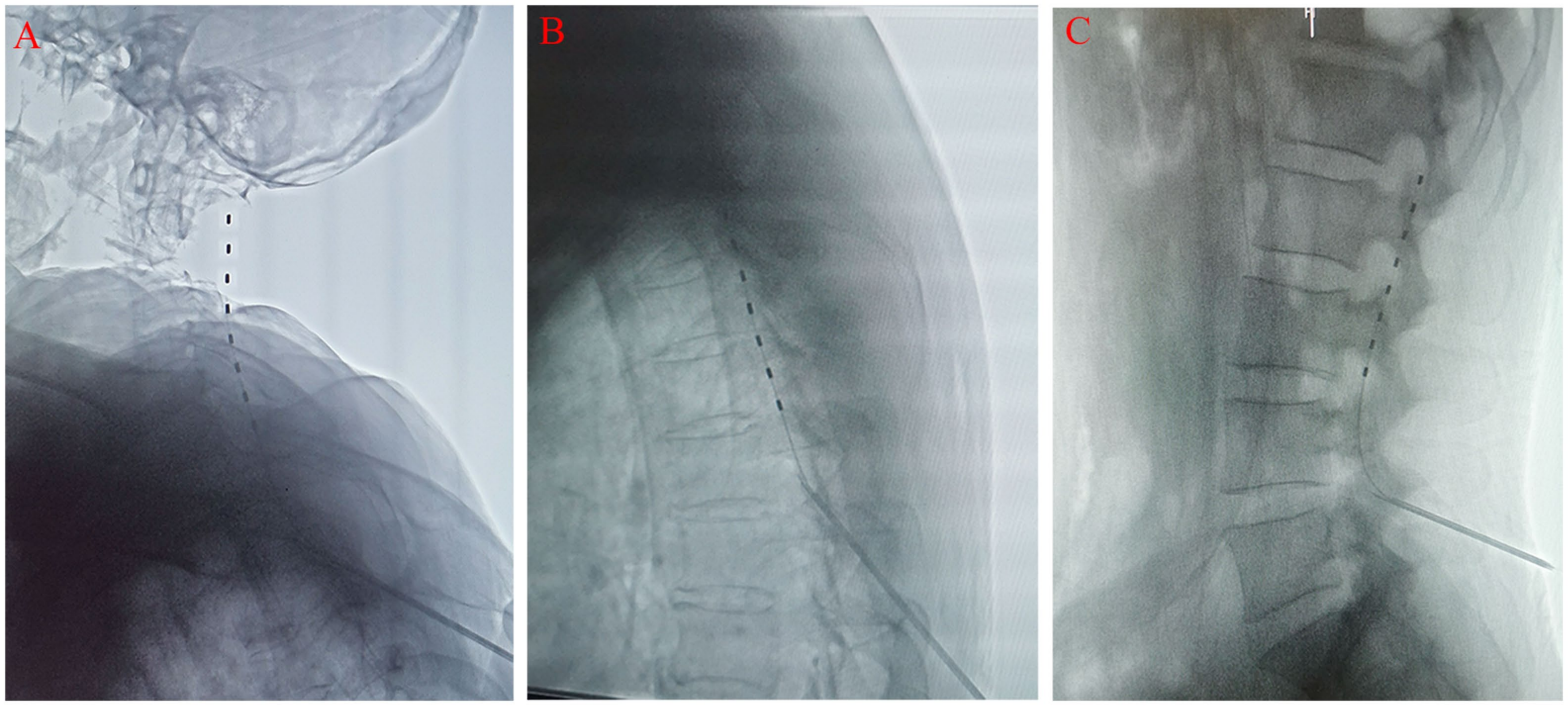
The lateral X-ray film shows that the electrode is located in the posterior epidural space. **(A)** neck; **(B)** chest; **(C)** lumbar.

Under the vigilant guidance of real-time DSA equipment, an 8-contact test electrode was delicately implanted into the epidural space. During this implantation, the orientation of the electrode was continuously and meticulously adjusted to optimize its positioning. The anteroposterior radiographic view clearly demonstrated that the electrode was accurately situated within the target area, lying outside the spinous process and inside the pedicle (Figure 3). Correspondingly, the lateral view affirmed its correct placement within the posterior epidural space (Figure 4).

Subsequently, the stimulator was connected, and the electrode contacts were fine-tuned based on the patient’s immediate responses. In parallel, parameters such as frequency (ranging from 60 to 240 Hz), pulse width (spanning 90 to 300 μs), and voltage (extending from 0.5 to 5.5 V) were adjusted to achieve the most efficacious therapeutic effect. The overarching objective was to ensure that the original herpes pain area was comprehensively blanketed by a perceptible tingling sensation. If deemed necessary, additional electrode contacts were activated to widen the coverage area of the tingling sensation, guaranteeing it exceeded that of the herpes pain region. Once optimal positioning was achieved, the electrode was securely fastened to the back skin.

In the postoperative phase, the patient was mandated to remain bedridden for 24 h to facilitate initial recovery. Regular dressing changes were scheduled, and close attention was paid to the puncture and fixation sites of the electrical stimulation for any signs of redness, swelling, heat, or pain. The electrical stimulation parameters were continuously adjusted in response to the patient’s feedback, ensuring maximum comfort and therapeutic benefit. Finally, after a continuous stimulation period exceeding 2 weeks, the electrode was carefully removed, concluding the treatment cycle.

### 2.4 Observation indicators and evaluation methods

The VAS scores of patients were meticulously collected and recorded prior to the surgical procedure, at the time of discharge, as well as during the follow-up evaluations conducted at 1 month, 3 months, and 6 months postoperatively. The follow-up process was implemented through a combination of outpatient revisitations, telephone interviews, and communication via WeChat. The VAS scoring system was employed to gauge the intensity of pain, wherein a score of 0 denoted the absence of pain, 10 signified excruciating pain, and scores ranging from 1 to 3 were indicative of mild pain, with the discomfort being sufficiently negligible so as not to impede sleep onset. Scores between 4 and 6 were representative of moderate pain, which had already begun to interfere with the patient’s sleep patterns. Scores from 7 to 9 corresponded to severe pain, rendering sleep attainment virtually impossible.

Based on whether the degree of pain alleviation in patients at the 6-month follow-up after surgery surpassed 50%, they were stratified into two cohorts: the Treatment Sensitive Group and the Treatment Insensitive Group. The computational formula for ascertaining the degree of pain relief was as follows: (VAS score 1 day before surgery-VAS score at follow-up) /VAS score before surgery. In cases where the degree of pain relief exceeded 50%, the patient was deemed responsive to stSCS treatment, signifying that the treatment had been efficacious and that the prognosis was promising. Conversely, if the relief degree was ≤ 50%, the patient was considered non-responsive to st-SCS treatment, indicating treatment inefficacy and a less favorable prognosis.

The utilization patterns of analgesic drugs (encompassing pregabalin, gabapentin, and other relevant pharmaceuticals) as well as the sleep conditions of patients throughout the follow-up period were meticulously recorded. To manage pain, oral pregabalin or gabapentin was administered, and the cumulative daily dosage during the follow-up phase was precisely documented. The sleep quality of patients was appraised using the Pittsburgh Sleep Quality Index (PSQI). The PSQI scale consists of seven principal constituents, specifically: subjective perception of sleep quality, time taken to fall asleep, total sleep duration, sleep efficiency, occurrence of sleep disturbances, the employment of sleeping pills, and the influence on daytime functioning. Each of these components was graded on a scale spanning from 0 to 3, with the overall score ranging between 0 and 21. Notably, a diminished total score is indicative of superior sleep quality.

The comprehensive data of patients were meticulously gathered, which incorporated a total of 23 variables as detailed in Table 1. Specifically, parameters such as age, gender, body mass index (BMI), the anatomic location of the affected area, side, course, preoperative VAS score, incidence of allodynia, extent of pain area, hypertension, diabetes mellitus, coronary disease, cutoimmune disorders, past medical history of tumors, utilization of immunosuppressive agents, leukocyte, hemoglobin level, monocyte count, C-reactive protein, erythrocyte sedimentation rate, hemoglobin A1, antiviral drug administration, and patient compliance were systematically recorded and subjected to in-depth analysis. Subsequently, a comparative assessment was performed to discern the disparities in the aforementioned case data between the two cohorts of patients. This was followed by a screening process to identify the potential risk factors that could impact the treatment outcome, culminating in the execution of Logistic regression analysis.

**Table 1:** Comparison of general characteristics between the two groups of patients.

Variables	Treatment sensitive group (*n* = 62)	Treatment insensitive group (*n* = 36)	Statistical value (*t*/*χ*^2^/*Z*)	*p* value
Age, Years (>60/≤60), [*n* (%)]	23/39 (37.1/62.9)	26/10 (72.2/27.8)	*χ*^2^ = 5.289	0.021^*^
Gender, (Male/Female), [*n* (%)]	30/32 (48.4/51.6)	17/19 (47.2/52.8)	*χ*^2^ = 0.012	0.911
BMI,(kg/m^2^,mean±SD)	23.8 ± 0.6	23.9 ± 0.7	*t* = −0.505	0.109
Location, [*n* (%)] (Cervical/Thoracic/Lumbar-Sacral)	10/30/22 (16.1/48.4/35.5)	6/18/12 (16.7/50.0/33.3)	*χ*^2^ = 2.547	0.280
Side (Left/Right), [*n* (%)]	28/34 (45.2/54.8)	16/20 (44.4/55.6)	*χ*^2^ = 0.113	0.737
Course (Days, mean±SD)	40.6 ± 11.9	86.5 ± 22.0	*t* = −13.350	0.000^*^
VAS score(mean±SD)	6.56 ± 1.10	6.33 ± 1.23	*t* = −0.970	0.365
Allodynia (Yes/No), [*n* (%)]	13/49 (21.0/79.0)	16/20 (44.4/55.6)	*χ*^2^ = 22.382	0.000^*^
Pain area (cm^2^, mean±SD)	116.4 ± 5.8	180.1 ± 14.3	*t* = −30.919	0.000^*^
Hypertension (Yes/No), [*n* (%)]	21/41 (33.9/66.1)	13/23 (36.1/63.9)	*χ*^2^ = 1.483	0.223
Diabetes mellitus (Yes/No), [*n* (%)]	9/53 (14.5/85.5)	17/19 (47.2/52.8)	*χ*^2^ = 15.813	0.000^*^
Coronary disease (Yes/No), [*n* (%)]	10/52 (16.1/83.9)	6/30 (16.7/83.3)	*χ*^2^ = 0.005	0.945
Autoimmune disorders (Yes/No), [*n* (%)]	5/57 (8.1/91.9)	11/25 (30.6/69.4)	*χ*^2^ = 8.004	0.004^*^
Tumor history (Yes/No), [*n* (%)]	4/58 (6.5/93.5)	9/37 (25.0/75.0)	*χ*^2^ = 5.335	0.021^*^
Immune suppressants (Yes/No), [*n* (%)]	3/59 (4.8/95.2)	8/28 (22.2/77.8)	*χ*^2^ = 6.907	0.009^*^
Leukocyte (×10^9^/L, mean±SD)	6.69 ± 1.41	7.17 ± 1.29	*t* = −1.649	0.323
Hemoglobin (g/L, mean±SD)	144.32 ± 11.05	120.05 ± 20.67	*t* = 6.034	0.000^*^
Monocyte (×10^9^/L, mean±SD)	0.48 ± 0.03	0.83 ± 0.04	*t* = −4.322	0.000^*^
C-reactive protein (mg/L, mean±SD)	6.48 ± 0.42	7.28 ± 0.35	*t* = −1.445	0.152
ESR (mm/h, mean±SD)	15.25 ± 2.09	17.77 ± 1.23	*t* = 1.286	0.096
HbA1 (%, mean±SD)	6.37 ± 1.88	7.49 ± 1.88	*t* = −2.925	0.004^*^
Antiviral medication (Yes/No), [*n* (%)]	56/6 (90.3/9.7)	27/9 (75.0/25.0)	*χ*^2^ = 43.513	0.000^*^
Compliance (Yes/No), [*n* (%)]	57/5 (91.9/8.1)	28/8 (77.8/22.2)	*χ*^2^ = 49.559	0.000^*^

According to the VAS score at the end of the follow-up (6 months after surgery), the degree of pain relief was calculated. Based on the degree of pain relief, the 98 enrolled patients were divided into two groups. There were 62 patients in the stSCS-sensitive treatment group (with a pain relief degree > 50%) and 38 patients in the stSCS-insensitive treatment group (with a pain relief degree ≤ 50%). The general data of the two groups were compared and analyzed. *P < 0.05.

BMI, Body mass index; ESR, erythrocyte sedimentation rate; VAS, visual analogue scale; HbA1,hemoglobin A1; stSCS, short-term spinal cord stimulation.

### 2.5 Statistical analysis

In the present study, a suite of software programs, namely IBM SPSS Statistics 26, GraphPad Prism 9.0, Microsoft Excel, and Adobe Photoshop CS6, were utilized for the purposes of data compilation, graphical illustration, and comprehensive statistical analysis. In the context of measurement data that adhered to a normal distribution pattern, normally distributed measurement data are presented as mean±SD, with the t-test being subsequently implemented for inferential analysis. Conversely, when the data deviated from a normal distribution, the median value, in conjunction with the quartile notation [M (P25, P75)], was adopted to characterize the dataset. For count data, a statistical description was effected through the use of frequencies expressed as percentages, and the chi-square test was invoked for hypothesis testing. In the case of measurement data pertaining to continuous variables subject to repeated measurements, repeated measures analysis of variance was engaged, and the Bonferroni test was employed for inter-group comparative evaluations. *p* < 0.05 was considered to indicate statistical significance.

In an endeavor to avert the potential collinearity problem among the variables under study, those indices manifesting statistically significant disparities in the univariate analysis were selected as variables. The data were also subjected to the Kaiser-Meyer-Olkin (KMO) test and Bartlett’s test of sphericity. The test results indicated that the data were suitable for principal component analysis (PCA). Thereafter, PCA was executed with the objective of reducing dimensionality, streamlining the data architecture, and extracting pivotal information. The determination of the number of principal components to be retained necessitated simultaneous compliance with the following principles (15, 16): (1) Based on cumulative contribution rate. Precisely, if the cumulative contribution rate of the initial K principal components reached 75%, then the first K principal components were retained. (2) Guided by eigenvalue magnitude. In other words, whenever the eigenvalue of a principal component surpassed 1, that particular principal component was preserved. Following the implementation of PCA, the screened independent variables were eligible for integration into the regression model, and a multivariate Logistic regression analysis was subsequently conducted with these incorporated independent variables. GraphPad Prism 9.0 was employed to devise the model, generate the calibration curve of the model, and further anticipate the model’s accuracy by computing the C-index. Moreover, the receiver operating characteristic (ROC) curve was meticulously drawn to prognosticate the sensitivity and specificity of the model at a more profound level and to meticulously assess the predictive efficacy of the devised model.

## 3 Results

### 3.1 Clinical efficacy

In this study, a total of 104 patients with ZAP were initially enrolled. However, 6 patients dropped out, and thus 98 cases were actually incorporated into the analysis. Among them, 62 patients were in the treatment sensitive group, and 36 patients were in the treatment insensitive group. The general information of the patients is presented in Table 1.

**Figure 5:**
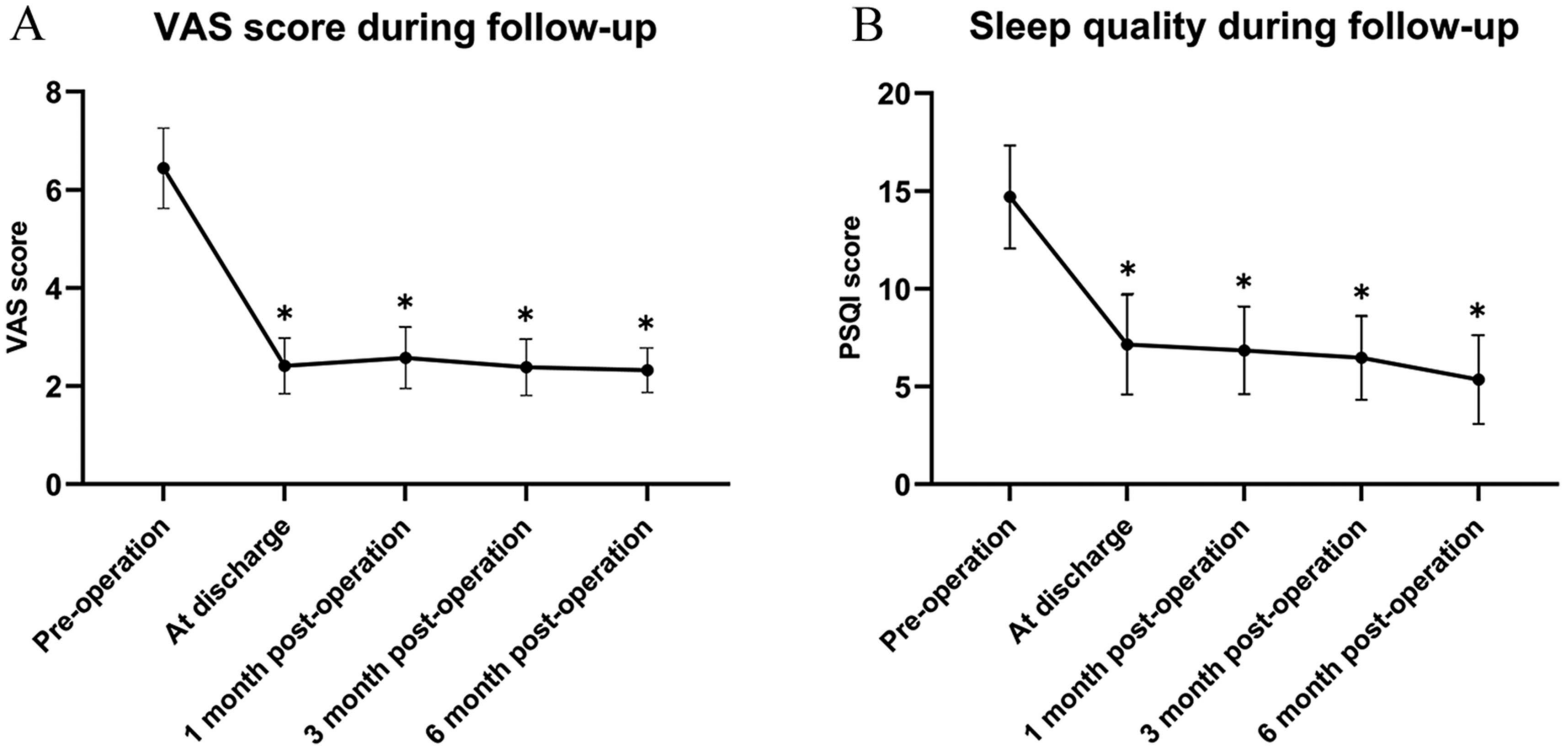
**(A)** Comparison of VAS; **(B)** Comparison of PSQI. Compared to before treatment, *p < 0.05. VAS, visual analogue scale; PSQI, Pittsburgh sleep quality index.

**Figure 6:**
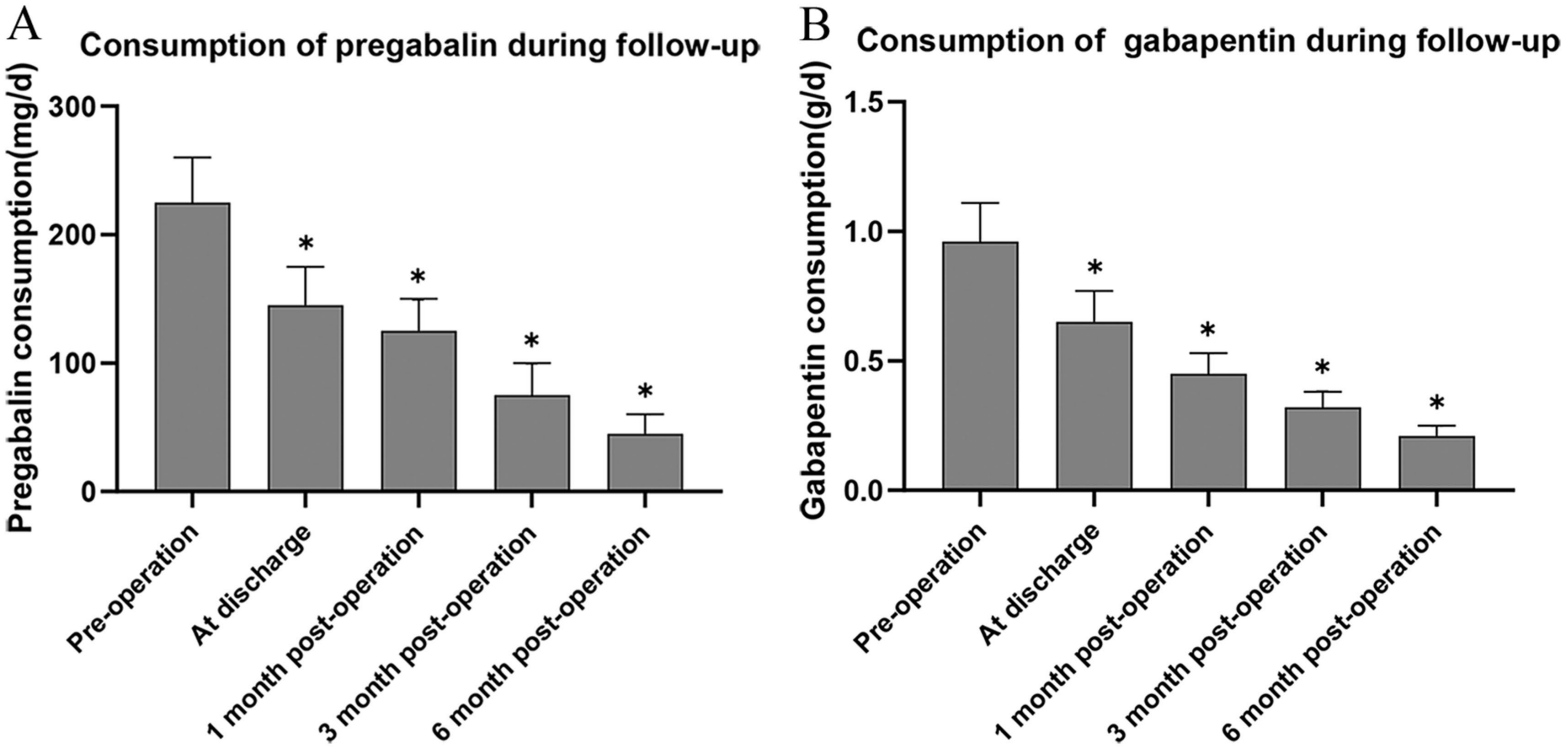
Mean consumption of pregabalin **(A)** and gabapentin **(B)**. Compared to before treatment. *p < 0.05.

Compared with the pre-treatment status, the VAS scores at each observation time point after stSCS treatment were significantly reduced (Figure 5A). The sleep quality of the patients was improved (Figure 5B), and the average dosages of pregabalin and gabapentin were markedly decreased compared with those before treatment (Figures 6A,B). The differences were statistically significant (^*^p < 0.05).

### 3.2 Complication

In accordance with the patient follow-up records, within the cohort of patients who underwent stSCS treatment, two instances of complications emerged during the hospitalization phase. To be precise, one case entailed electrode displacement, while the other manifested as a mild cerebrospinal fluid leak. For the patient experiencing electrode displacement, during the hospital stay, subsequent to the meticulous adjustment of other electrode contacts and stimulation parameters, the pain region of the patient was predominantly encompassed by the effect of spinal cord stimulation. Upon achieving this, the patient was deemed fit for discharge. In the scenario of the patient with a mild cerebrospinal fluid leak, a series of symptomatic and supportive interventions were implemented, such as enforced bed rest and the provision of ample fluids and electrolytes to maintain physiological balance. The patient did not exhibit any conspicuous discomfort and was discharged once the symptoms had abated and the condition had reached a stable state. Throughout the follow-up period, no further complications were detected.

### 3.3 Prognostic factor analysis

In combination with the patients’ clinical characteristic data, the results of univariate analysis indicated that factors such as gender, BMI, location, laterality, preoperative VAS score, hypertension, coronary disease, leukocyte, C-reactive protein, and erythrocyte sedimentation rate exhibited no significant correlation with the treatment outcome after stSCS surgery in patients with ZAP (*p* > 0.05). In contrast, for the patients in the treatment sensitive group, indicators including age, course, allodynia, pain area, coexistence of diabetes mellitus, autoimmune diseases, tumors, use of immunosuppressants, hemoglobin, monocyte count, HbA1, antiviral drug use, and compliance showed significant correlations with those in the treatment insensitive group (*p* < 0.05) (Table 1).

**Figure 7:**
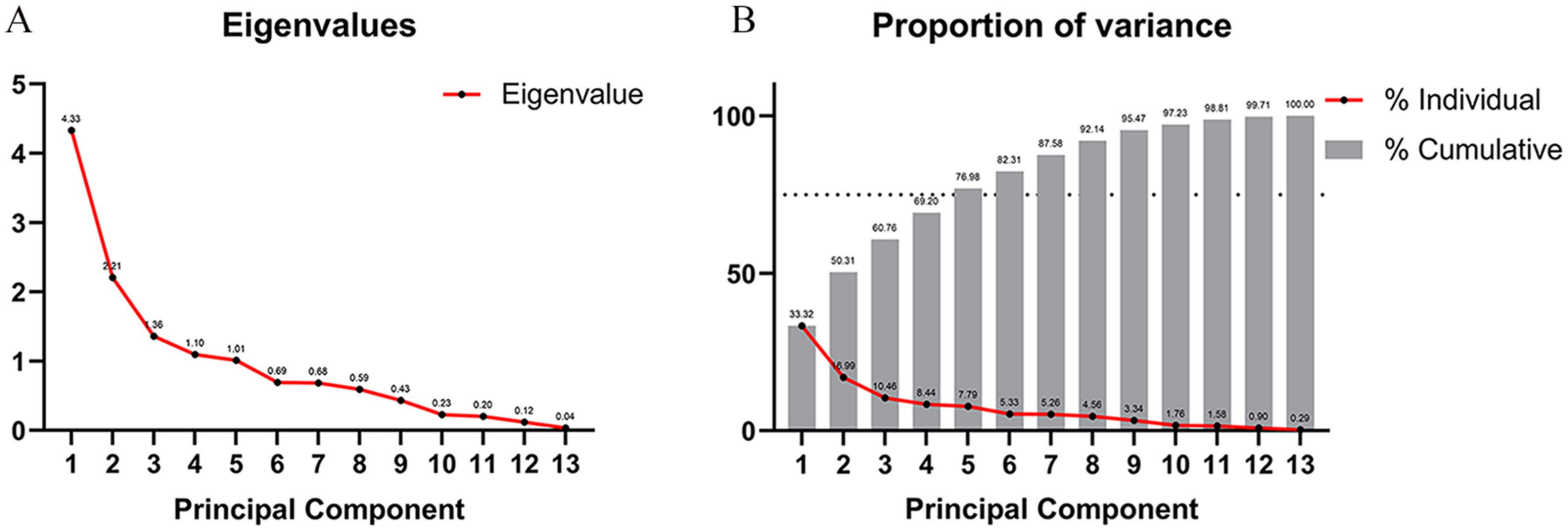
Principal component analysis and determination of the number of variables. **(A)** Scree plot, starting from the fifth principal component, the eigenvalue of the principal component begins to decline slowly, indicating that the first five principal components can explain most of the variance of the data. The contribution of subsequent principal components to the variance gradually decreases. We select principal components with eigenvalues greater than 1. **(B)** Proportion of variance lot, when the cumulative contribution rate of the principal components that can be explained reaches 75%, we select the first five principal components.

In an effort to evade the collinearity issue plaguing the variables within this study, PCA was executed on those independent variables that displayed significant correlations between the two groups of patients. The determination of the appropriate quantity of principal components was achieved through the employment of the Scree Plot and the Proportion of Variance Plot. The outcomes gleaned from the Scree Plot (Figure 7A) signified that, commencing from the 5th principal component, the eigenvalue of the principal components embarked on a gradual downward trend. This implies that the foremost 5 principal components were proficient in accounting for the lion’s share of the variance present in the data, while the contributions of the subsequent principal components to the variance progressively tapered off. As a result, the principal components possessing eigenvalues greater than 1 were cherry-picked. The results furnished by the Proportion of Variance Plot (Figure 7B) evinced that, provided that the cumulative contribution rate of the principal components hit 75%, the first 5 principal components were handpicked to ascertain that these principal components could adeptly encapsulate the essential information of the original variables. By synthesizing the revelations of both plots, it was conclusively determined that age, course, allodynia, pain area, and a history of diabetes mellitus constituted the five principal components wielding an influence on the prognosis.

### 3.4 Model prediction and evaluation

**Figure 8:**
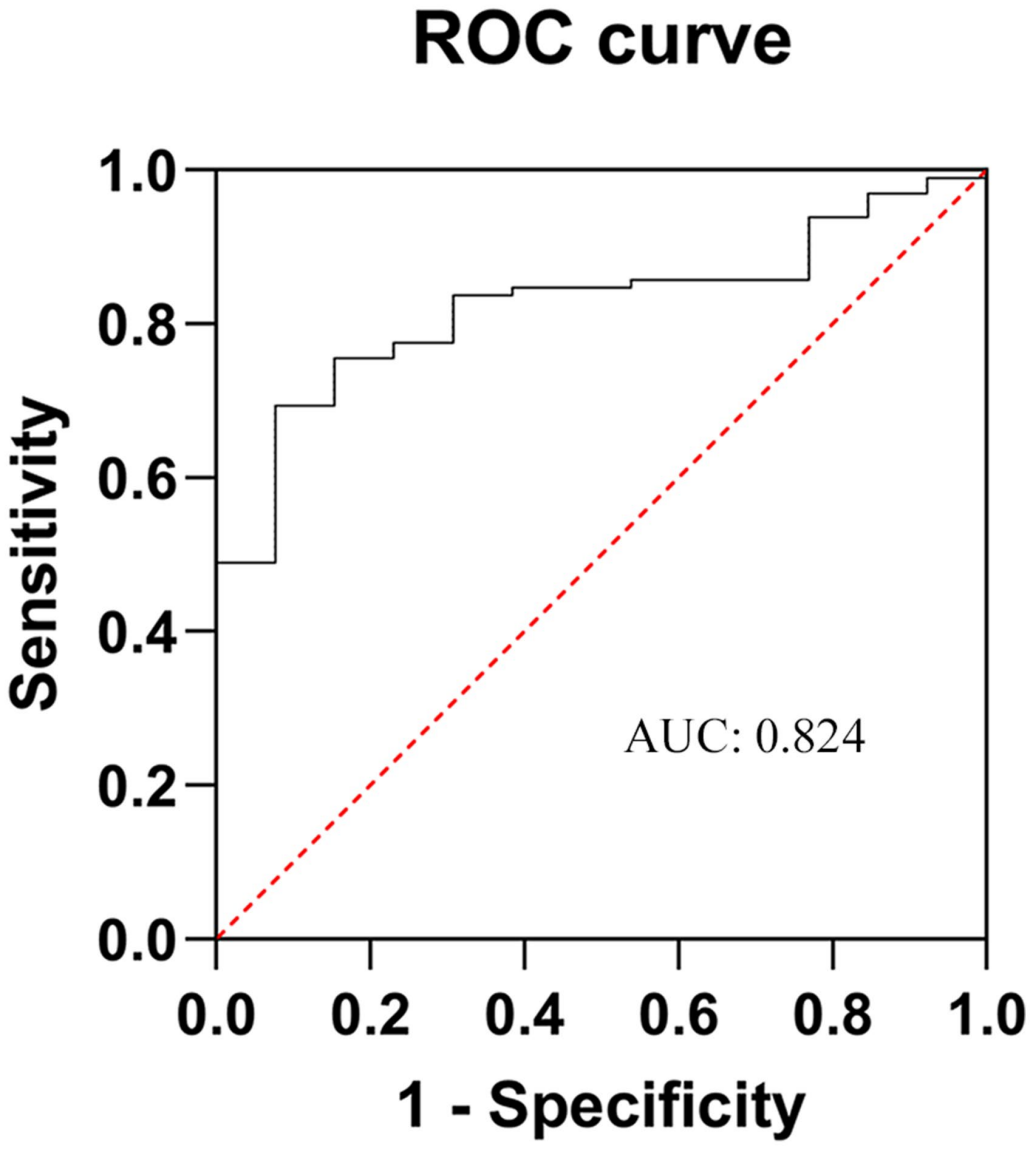
ROC curve of the poor prognosis prediction model for ZAP patients after stSCS. AUC is area under the curve. The closer it is to 1, the higher the prediction accuracy of the model. ZAP, zoster-associated pain; stSCS, short-term spinal cord stimulation.

The above-mentioned five factors were then placed under multivariate regression analysis by means of GraphPad Prism 9.0, and the relevant numerical values were fed into the Logistic regression model for comprehensive examination. The resultant data divulged (Table 2) that age [odds ratio (OR): 1.175; 95% confidence interval (CI): 1.864–2.584; *p* = 0.021], disease course (OR:1.894; 95% CI: 1.563–2.365; *p* = 0.003), diabetes mellitus (OR: 2.805; 95% CI: 2.425–3.539; *p* = 0.025), and the pain area size (OR: 3.208; 95% CI: 1.705–2.213; *p* = 0.001) were independent risk determinants that bore a significant influence on the therapeutic efficacy of stSCS. The computed C-index stood at 0.824, and the area under the receiver operating characteristic curve (ROC), referred to as AUC, was quantified as 0.824 (95% CI, 1.761–3.278), as depicted in Figure 8.

**Table 2:** The results of logistic regression analysis.

Variables	Regressioncoefficient (β)	Standard error (SE)	Odds ratio (OR)	95% confidence interval (CI)	*P* value
Age	0.582	0.151	1.175	1.864–2.584	0.021^*^
Course	0.497	0.224	1.894	1.563–2.365	0.003^*^
Allodynia	2.697	0.216	1.546	1.348–1.824	0.079
Pain Area	0.658	0.286	3.208	1.705–2.213	0.001^*^
DiabetesMellitus	1.672	0.321	2.805	2.425–3.539	0.025^*^

Conduct Logistics regression analysis on the variables screened by principal component analysis. *P < 0.05.

## 4 Discussion

Zoster-associated pain (ZAP) constitutes one of the most commonly encountered and gravely troublesome complications stemming from HZ. It subjects patients to intense agony and takes a heavy toll on their quality of life. Traditional treatment approaches, including pharmacotherapy and nerve block, have proven to be less than satisfactory in terms of efficacy when it comes to certain patients afflicted with refractory ZAP. This has, in turn, galvanized continuous endeavors within the clinical realm to seek out more potent and effective therapeutic alternatives. Short-term spinal cord stimulation (stSCS), being a secure and efficacious neuromodulation technique, has witnessed a progressively expanding scope of application in the management of ZAP (17).

One of the primary mechanisms by which stSCS functions in the treatment of ZAP lies in its ability to impede the transmission of pain signals. This principle is rooted in the “Gate Control Theory” put forward by Melzack and Wall in 1965 (18). During the implementation of stSCS, electrodes are meticulously inserted into the epidural space of the corresponding spinal segments within the vertebral canal. Subsequently, the pulsed current discharged by these electrodes can effectively interact with the spinal nerve fibers. By activating the excitability of inhibitory interneurons in the spinal cord, it prompts the Aβ fibers to diminish the ascending pain signals, thereby disrupting the conduction pathway of pain signals and accomplishing pain relief (19). Furthermore, stSCS, when acting on the posterior columns of the spinal cord, augments the release of *γ*-aminobutyric acid (GABA), triggers an elevation in serotonin levels, and to a variable extent, promotes the secretion of endogenous analgesic substances (20). In our clinical practice, the range of 60-240 Hz allows for fine-tuning based on the patient’s pain level, nerve injury site, and treatment response to ensure optimal efficacy. Research findings suggest that spinal cord stimulation can also exert its influence by upregulating the expression of brain derived neurotrophic factor (BDNF), fostering local neural plasticity, and engaging in the modulation of glial cells and neuroinflammation (21). In addition, stSCS holds the potential to enhance nerve function. It achieves this by modulating the synaptic connection strength among neurons, facilitating the generation of new synapses, as well as regulating neuronal excitability and neural plasticity (22). As result, it alleviates pain and expedites the restoration of sensory function. This aspect is of crucial significance for the long-term amelioration of nerve function in ZAP patients and furnishes a prospective neurobiological foundation for augmenting the quality of life of these individuals.

Our investigation manifested that, when contrasted with the pre-treatment condition, patients attained substantial alleviation of pain (as clearly manifested by a pronounced diminution in VAS scores) at diverse follow-up junctures subsequent to stSCS treatment. Concomitantly, an amelioration in sleep quality (evinced by a decline in the PSQI) was observed, along with a reduction in the mean dosage of analgesic pharmaceuticals (pregabalin and gabapentin). In parallel, copious prior clinical investigations have signified that stSCS presents conspicuous merits over conventional treatment modalities in respects of the magnitude of pain relief, the persistence of therapeutic efficacy, and the prophylaxis of PHN onset (23, 24). This unequivocally attests that stSCS endows patients with a more reliable and enduring pain abatement outcome, thereby playing a pivotal role in augmenting patients’ quality of life. Notwithstanding the fact that stSCS has evinced a certain degree of efficacy in the management of ZAP, it may nonetheless be attended by several complications. Antecedent research has divulged that electrode displacement, infection, epidural hematoma, cerebrospinal fluid leakage, and nerve injury constitute prevalent complications affiliated with this technique (25). During our treatment regimen, one instance of mild electrode displacement and one instance of mild cerebrospinal fluid leakage transpired. By virtue of our profound comprehension of these complications and the prompt and efficacious intervention, the safety profile of stSCS treatment was fortified, curtailing patients’afflictions and risks and guaranteeing the seamless progression of the treatment as well as the prognostic caliber of the patients.

Although stSCS can effectively mitigate the pain level of patients with ZAP and improve their sleep quality, there remains a subset of patients who experience suboptimal pain relief, with recurrent symptoms and poor therapeutic outcomes. Consequently, analyzing the influencing factors of stSCS in the treatment of ZAP, implementing early targeted interventions for ZAP, optimizing treatment regimens, and enhancing the success rate of surgeries are of paramount importance. In this study, a prediction model for the ineffectiveness of stSCS treatment was established. The results of univariate analysis, PCA, and multivariate regression analysis indicated that age, course, coexistence of diabetes mellitus, and the area of the pain region were four independent risk factors influencing the efficacy of stSCS.

Age, being a crucial risk determinant, mirrors the holistic physiological condition of the organism, the potency of its immune system, and the extent of functional degeneration. As time elapses and age advances, a progressive decline becomes evident in the diverse physiological functions of the body. Concomitantly, the reparative capabilities of the nervous system and its proficiency in pain modulation wane. This is exemplified by a diminished neuron count, modified neurotransmitter metabolism, and decelerated nerve fiber conduction velocity (26). Such alterations potentially bear upon the onset and progression of ZAP and, moreover, influence the responsiveness to stSCS treatment (27). In a follow-up study involving 272 patients afflicted with ZAP, 59 individuals developed PHN, yielding a prevalence rate of 21.7%. Notably, those subjects harbouring PHN, with an average age of 70.9 years, were considerably older than their counterparts without such a condition, whose average age stood at 64.2 years (28). This disparity could plausibly be ascribed to the enfeebled immune function and the subpar regenerative and restorative capacities of nerve tissues characteristic of elderly patients. Our research findings disclosed that within the treatment sensitive group, the percentage of patients exceeding 60 years of age was 37.1%, whereas in the treatment insensitive group, this figure soared to 72.2%. These revelations imply that, in the realm of clinical practice, for geriatric patients, a meticulous and comprehensive evaluation of their physical well-being and neurological functions ought to be carried out pre-treatment. Subsequently, more personalized treatment blueprints need to be devised, potentially entailing judicious adjustments to the electrical stimulation parameters or the incorporation of supplementary adjunctive treatment modalities, all with the aim of augmenting the likelihood of a successful treatment outcome.

The length of the disease course is intimately tied to the treatment effectiveness. Patients with a protracted disease course typically exhibit more pronounced nerve damage. The restructuring of the pain signal conduction pathway becomes increasingly complex, and the more extended the period during which the nerves are exposed to viral encroachment and inflammatory reactions, the more severe the degenerative, necrotic, and demyelinating alterations of the nerve fibers are likely to be (29, 30). These modifications potentially pose a challenge for stSCS in fully reversing the pre-existing pathological changes, consequently impinging on the treatment outcome. A research study illustrated that among 99 patients with ZAP who underwent stSCS, over the course of a 12-month follow-up, merely 2.5% of the patients in the AHN group endured persistent pain necessitating analgesia, 16.0% in the SHN group, and 62.5% in the PHN group (31). This finding indicates that the early employment of stSCS can efficaciously forestall the onset of PHN. Our own research findings disclosed that the disease course of patients within the treatment sensitive group averaged (40.6 ± 11.9) days, whereas that of patients in the treatment insensitive group was (86.5 ± 22.0) days. This implies that for patients with a more prolonged disease history, early intervention is of paramount significance. The timely initiation of st-SCS treatment during the acute or subacute stage of the ailment might potentially culminate in more favorable results.

Diabetes mellitus has been shown to precipitate neuropathy. In a hyperglycemic milieu, metabolic derangements transpire within nerve cells, giving rise to impairments in the structural and functional integrity of nerve fibers and curtailing the nerves’ reactivity to electrical stimulation. Concurrently, diabetes can also impinge on blood circulation, diminishing the nutrient provision to nerve tissues and further compounding nerve damage (32). Inadequate glycemic regulation not only exacerbates pain manifestations but also attenuates the nerves’ responsiveness to electrical impulses, thereby undermining the therapeutic efficacy of stSCS (33). A decade-long observational study divulged that diabetes substantially augmented the hospitalization rate, readmission rate, and the susceptibility to complications in relation to HZ. Among diabetic patients, the risk factors for hospitalization due to HZ encompassed an age exceeding 65 years, obesity, and suboptimal glycemic control (HbA1c > 8.0%). Following HZ, the readmission risk for these patients surged by 40% (34). Our research findings evinced that the proportion of diabetic patients within the treatment sensitive group was 14.5%, whereas that in the treatment insensitive group was 47.2%. Hence, for ZAP patients with concurrent diabetes, vigorous glycemic control measures ought to be instituted to mitigate neuropathy. Simultaneously, vigilant surveillance of nerve function alterations is imperative, and prompt modification of the treatment protocol is requisite to attain satisfactory clinical treatment endpoints for patients.

Within the framework of ZAP, the pain area chiefly denotes the expanse where pain symptoms emerge in the nerve innervation territory invaded by the VZV. The appraisal of the pain area assumes pivotal importance in discerning the gravity of the condition and appraising the potency of the treatment (35). A broader pain area indicates a more extensive reach of the affected nerves, which presumably corresponds to a more severe level of nerve deterioration. Simultaneously, it heightens the intricacy for stSCS to thoroughly cover the pain area and adeptly modulate nerve function, ultimately culminating in a diminished treatment outcome. Our research revelations disclosed that the pain area of patients in the treatment sensitive group measured (116.4 ± 5.8) cm^2^, while that of patients in the treatment insensitive group amounted to (180.1 ± 14.3) cm^2^. Thus, for patients displaying allodynia and harboring a relatively sizable pain area, the concurrent utilization of drug therapy or other physical treatment modalities during the treatment course could be considered, with the objective of augmenting the curative efficacy of stSCS.

The C-index is utilized to assess the level of concordance between the projected outcomes of a model and the actual observational findings, with its value span falling within the range of 0.5 to 1. As it draws nearer to 1, the predictive precision of the model escalates. The area under the ROC curve, abbreviated as AUC, constitutes a vital metric for appraising the accuracy and discriminatory prowess of a model. More precisely, AUC graphically and vividly depicts, via the ROC curve, the sensitivity and specificity of the model at diverse threshold settings (36). A greater AUC value implies that the model is more proficient at differentiating between patients who respond positively to treatment and those who do not, thereby denoting enhanced predictive capabilities. In the current study, the computed C-index amounted to 0.824, and the AUC was 0.824 (95% CI, 1.761–3.278), intimating that the model demonstrates commendable predictive aptitude and accuracy.

## 5 Limitations

Our study also suffers from several limitations, including a relatively small sample size, a single-center retrospective design, and a comparatively short follow-up duration. In addition, regarding the potential limitation of the clinical interpretability of the principal components, in our subsequent research, we plan to conduct a more in - depth analysis of the principal components by integrating clinical expertise and relevant literature. In the future, it is imperative to conduct randomized controlled trials with larger sample sizes and in a multi-center setting to further verify the long-term efficacy and safety of stSCS treatment.

## 6 Conclusion

In conclusion, stSCS effectively relieves ZAP patients’ pain, reduces analgesic consumption, and improves sleep quality, with low complication incidence and high safety. Notably, age, disease course, diabetes mellitus, and pain area extent are independent risk factors for stSCS efficacy. The computed C-index and ROC curve area were both 0.824, indicating the prediction model’s good accuracy and discriminative power, which has significant clinical guiding significance, helping physicians identify suitable patients and formulate individualized treatment plans.

## Data Availability

The original contributions presented in the study are included in the article/supplementary material, further inquiries can be directed to the corresponding author/s.

## Glossary

AHN: Acute herpetic neuralgia
AUC: Area under curve
BDNF: Brain derived neurotrophic factor
BMI: Body mass index
CI: Confidence interval
DSA: Digital subtraction angiography
ESR: Erythrocyte sedimentation rate
GABA: *γ*-aminobutyric acid
HbA1: Hemoglobin A1
HZ: Herpes zoster
PCA: Principal component analysis
PHN: Postherpetic neuralgia
PRF: Pulsed radiofrequency
PSQI: Pittsburgh sleep quality index
ROC: Receiver operating characteristic
SHN: Subacute herpetic neuralgia
stSCS: Short-term spinal cord stimulation
VAS: Visual analogue scale
VZV: Varicella-zoster virus
ZAP: Zoster-associated pain
